# Twins Assessing Their Own and Parental Intelligence: Examining the Raters’ Agreement and the Effect of Raters’ and Targets’ Gender

**DOI:** 10.5964/ejop.v16i2.1853

**Published:** 2020-05-29

**Authors:** Denis Bratko, Martina Pocrnić, Ana Butković

**Affiliations:** a Department of Psychology, Faculty of Humanities and Social Sciences, University of Zagreb, Zagreb, Croatia; University of Wroclaw, Wroclaw, Poland

**Keywords:** self-assessed intelligence, other-assessed intelligence, Gardner’s multiple intelligences, twin study, gender

## Abstract

The goal of this study was to explore the raters’ agreement and the effect of raters’ and targets’ gender on self- and parental intelligence assessments in the sample of Croatian twins. Twins were asked to assess their own and their parents’ overall intelligence, as well as specific abilities from the Gardner’s theory of multiple intelligences. Data was analysed to explore: i) twins’ agreement in parental assessments and behavioural genetic analysis of the overall intelligence estimates; ii) gender differences in self- assessments; and iii) raters’ and targets’ gender effects on parental assessments. The twins’ mean correlation in their assessments of overall parental intelligence was .60. The differences between monozygotic and dizygotic twin correlations were nonsignificant for all of the estimated abilities, and model fitting analysis indicates that hypothesis about genetic effect on parental assessment of intelligence should be rejected. The hypotheses about males’ higher self-assessments for overall intelligence and for the masculine types of abilities - logical-mathematical, body-kinesthetic and spatial abilities - were confirmed. For the feminine types of abilities - verbal/linguistic, inter- and intra- personal intelligences - there were no significant gender effects. Both target and rater effect were found for the parental estimates of intelligence. Fathers were estimated higher on overall intelligence, logical-mathematical, body-kinesthetic and spatial abilities, while mothers were estimated higher on interpersonal and intrapersonal intelligence. The effect of the raters’ gender was found for overall intelligence as well as for inter- and intra- personal intelligences, where males gave higher estimates of parental intelligences than females.

Intelligence is one of the central concepts in differential psychology. Numerous psychometrically validated tests were developed for assessing one's intelligence score. However, in daily life lay people often evaluate their own abilities, as well as abilities of others without using standardized measures. These self- and other- assessments of intelligence (SAI and OAI) can be detected by asking individuals to estimate their own intelligence quotient (IQ) or IQ of others on a bell curve of intelligence with a mean of 100 and a standard deviation of 15, using a deviation IQ scale as a model. Obviously, impressions about self- and other- intelligence are not only based on the actual abilities. Recent meta-analysis indicates that constructs which correlate with SAI can be categorized into four groups: i) constructs associated with intelligence; ii) tendencies and opportunities to develop intelligence; iii) constructs associated with biased self- assessments; and iv) positive states and life achievements (Howard & Cogswell, 2018).

## Association Between Subjectively Assessed and Measured Intelligence

Although one can assume that people are able to assess their own and others’ intelligence quite precisely, studies have shown that the correlation between SAI and psychometric intelligence is usually around .30, with range between .20 and .50 (Ackerman & Wolman, 2007; Furnham, 2001). Bratko, Butkovic, Vukasovic, Chamorro-Premuzic, and von Stumm (2012) have found correlation of .33 between measured IQ and SAI in the sample of Croatian twins. Borkenau and Liebler (1993) investigated the convergence between measured and subjectively assessed IQ. They found that measured intelligence correlates .32 and .29 with self- and acquaintance ratings of intelligence, respectively. These findings indicate that SAI and OAI are not very precise and that they certainly cannot replace the validated measures of intelligence. In order to explain inaccuracy of SAI, researchers hypothesized that they may occur due to the lack of metacognitive insight or due to the broadness and the ambiguousness of the overall intelligence as a trait being evaluated (Freund & Kasten, 2012). The similar reasoning regarding broadness and ambiguousness of the intelligence concept might be extrapolated to OAI as well. The Realistic Accuracy model borrowed from personality judgments (Funder, 1995; see also Connelly & Ones, 2010) describes accuracy as a function of the availability, detection, and utilization of relevant behavioral cues, and recognition of these cues might be relevant for the accurracy in assessing abilities of other people as well.

## The Twin Studies of Subjectively Assessed Intelligence

Classical twin study compares phenotypic resemblances of monozygotic (MZ) twins who are genetically identical and dyzgotic (DZ) twins who share half of their genes on average in order to estimate the extent to which genetic variation contributes to the phenotypic variation of the trait. Meta-analysis of twin correlations and reported variance components for 17,804 traits showed that the heritability estimate across all traits is 49% (Polderman et al., 2015). There are only a few twin studies of SAI and they have shown that heritability of self-assessed abilities is as high as for measured intelligence. Spinath, Spinath, and Plomin (2008) reported genetic influences of 40%, Greven, Harlaar, Kovas, Chamorro-Premuzic, and Plomin (2009) reported heritability of 51%, while Bratko et al. (2012) reported heritability of 57%. On the other hand, as far as we know, there are no prior twin studies, which include OAI. Twin design in estimating parental intelligence can also allow, besides testing the hypothesis about genetic and environmental influence, the investigation of rater agreement in OAI. We know little about the agreement of family members in their estimation of abilities of other family member. For example, in a broad personality domain, the meta-analysis (Connelly & Ones, 2010) yielded rater-rater correlations of two family members estimating other family member traits of .37, .45, .38, .25, and .36 for Neuroticism, Extraversion, Openness, Agreeableness, and Conscientiousness, respectively. Although we are not aware of such family rater-rater agreement studies of OAI, Borkenau and Liebler (1993) reported average correlation of .52 between self- and close acquientance intelligence estimate.

## The Hubris-Humility Effect

Even though SAI and OAI are not fully correspondent with psychometrically measured intelligence, these estimates are interesting research topic because they can provide important information about the lay views of intelligence. In general, studies have shown that people are likely to overestimate their intelligence score and see themselves as above the average. However, there is a difference between men and women in that trend. Namely, men usually provide statistically higher estimations than women do. That was firstly stated in Hogan's (1978) pioneer study of intelligence estimation where he reported that in half of his 11 studies the difference between male and female intelligence self-estimations was statistically significant. Since then, there have been a number of studies worldwide confirming that trend (e.g. Beloff, 1992; Bennett, 1996, 1997, 2000; Furnham & Chamorro-Premuzic, 2005; Furnham, Kosari, & Swami, 2012; Furnham & Storek, 2017; Neto, Furnham, & da Conceição Pinto, 2009; Kang & Furnham, 2016; Rammstedt & Rammsayer, 2000; von Stumm, Chamorro-Premuzic, & Furnham, 2009). This gender difference in intelligence self-estimation is considered consistent and culturally invariant and is known as hubris-humility effect (Furnham, Hosoe, & Tang, 2001).

Besides self-estimations of overall intelligence, many studies investigated gender differences in specific domains, mostly using Gardner's (1983, 1999) theory of multiple intelligences. Gardner (1983) initially suggested that every individual should develop to some extent seven different types of intelligence. He defined ''object-related'' forms that include logical-mathematical, spatial and body-kinesthetic intelligence. The ''object-free'' forms consist of verbal/linguistic and musical intelligence. Finally, there are two types of personal intelligence, interpersonal and intrapersonal domains. Lately, Gardner (1999) defined three new possible types – naturalistic, spiritual, and existential intelligence, but concluding that only naturalistic intelligence merits addition to seven original types. Although Gardner’s theory is not a psychometrically validated theory of the intelligence structure, and Gardner has not designed instrument for measuring his different types of intelligence, it has been extensively used in the study of SAI. The reason for this was that this theory offered a more fine-grained analysis of the differences in lay self-estimates, and not because the idea was to validate or support the theory itself (Furnham, 2001). In addition, Gardner’s view of intelligence is very appealing to lay people due to its view that everyone can be high in some type of intelligence. Studies that investigated self-estimations of these multiple intelligences showed that the biggest and most consistent differences between men's and women's self-estimates were found in mathematical/logical and spatial intelligence, with men providing higher scores. Similar pattern was also found in the studies that used psychometric Cattell-Horn-Carroll theory of intelligence (Ortiz, 2015) with men giving higher scores than women in estimating their overall intelligence and the most of broad abilities (Furnham & Mansi, 2014; Kang & Furnham, 2016). A meta-analytic study by Syzmanowicz and Furnham (2011) found the highest effect-size, with males assessing themselves higher than females, for mathematical/logical (*d* = .44), followed by spatial (*d* = .43) and overall (*d* = .37) intelligence, while the smallest effect size was in verbal/linguistic (*d* = .07) SAI where females provided higher estimates.

## Gender Effect in Intelligence Assessment of Others

Although most of the studies focused on the people's belief about their own intelligence, some of them also showed that these gender differences were not limited to SAI. Studies of OAI indicate that there is a noticeable trend where people see their fathers, grandparents or sons as more intelligent than their mothers, sisters and daughters (Furnham, 2000; Furnham & Chamorro-Premuzic, 2005; Furnham & Gasson, 1998; Furnham et al., 2001; Neto & Furnham, 2011). Given that the focus of this study, besides gender differences in SAI, are gender differences in OAI or, more specifically, the parental estimates, we will also review the findings in that field. In his pioneer study, Hogan (1978) also reported that both male and female students attributed higher IQ scores to their fathers than to their mothers. That was followed by Beloff's (1992) finding that women believe they are intellectually equal to their mothers, but inferior to their fathers, while men saw themselves as equal to their fathers and superior to their mothers. Since then, there have been several studies in different cultures showing that people tend to give higher overall intelligence scores to their fathers than to their mothers (e.g. Furnham & Chamorro-Premuzic, 2005; Furnham & Wu, 2008; Furnham et al., 2012; Petrides, Furnham, & Martin, 2004; Swami, Furnham, & Zilkha, 2009). Along with overall intelligence, studies also focused on perceived differences in parents' multiple intelligences. As well as with self-estimations, the most consistent finding is that fathers are believed to be more intelligent in mathematical/logical and spatial domains (Furnham & Wu, 2008; Ortner, Müller, & Garcia-Retamero, 2011; Swami et al., 2009). On the other hand, mothers are often given higher scores on interpersonal, intrapersonal and musical intelligence (Bennett, 1996; Ortner et al., 2011; Rammstedt & Rammsayer, 2000). These findings indicate division of masculine and feminine types of abilities. Bennett (2000) reported that people perceive mathematical, spatial and body-kinesthetic intelligences as more masculine, while personal, musical, and verbal/linguistic intelligences are perceived as abilities that are more feminine. Interestingly, in some studies fathers were given higher estimates in verbal factor (Furnham et al., 2012; Rammstedt & Rammsayer, 2000; Swami et al., 2009), although it is usually considered as type of ability where women excel. In addition, Furnham (2000) suggests that people primarily relate IQ to mathematical and spatial domains and see them as essence of intelligence. Therefore, it can be said that people see intelligence as male-normative. Indeed, studies have shown that the best predictors of estimated overall intelligence are numerical and spatial domains (Furnham, 2001; Furnham et al., 2012; Neto & Furnham, 2011; Swami et al., 2009). This illustrates that people do not have a broad view on intelligence that includes all domains suggested by Gardner, but that they are mostly focused on masculine types of abilities when considering overall intelligence.

## Sources of Gender Differences

There are two different hypotheses that have been discussed in order to explain gender differences in estimated intelligence. First one argues that gender differences in estimations reflect small but real differences in intelligence between men and women which people tend to overestimate. Thus, people estimate men as more intelligent because they observe that pattern in daily lives (Furnham & Rawles, 1995). However, results are inconclusive because gender differences in overall intelligence have been found in both the direction of higher men’s and higher women’s scores (e.g. Daseking, Petermann, & Waldmann, 2017; Irwing, 2012; Keith, Reynolds, Patel, & Ridley, 2008; Reynolds, Keith, Ridley, & Patel, 2008; van der Linden, Dunkel, & Madison, 2017), while some research shows that there are negligible gender differences in overall intelligence (e.g. Camarata & Woodcock, 2006; Flynn & Rossi-Casé, 2011; Halpern, 2012; Savage-McGlynn, 2012; Sternberg, 2014). Psychometric differences are usually found in the mathematical/logical and spatial domain where men have higher results (Hyde, Fennema, & Lamon, 1990; Voyer, Voyer, & Bryden, 1995), which is in line with gender differences in multiple intelligence estimations.

The second hypothesis states that gender differences can primarily be attributed to gender stereotypes (Rammstedt & Rammsayer, 2000). When it comes to parental estimations, judgments about their IQ score could also be associated with mothers' and fathers' family roles, work status or other additional cues which raters might use. Ortner et al. (2011) showed that estimation of parents' overall intelligence can be predicted by their education and current employment status and argue that being unemployed or working part-time is more related to feminine gender role, while full-time employment is mostly perceived in relation to the male gender role and thus the higher estimations of intelligence.

## Rater Effect

Another phenomenon in OAI are possible differences in estimates between male and female raters. Thus, the question is does hubris-humility effect extend to estimates of the others – both male and female targets? If males tend to give themselves higher estimations than females do, does that mean they are also more prone to give higher estimates to all others? Certain studies have confirmed that assumption. For example, in Swami et al. (2009) study males rated both of their parents as having higher intelligence for most intelligence types than did females, and Furnham et al. (2001) reported that male participants rated fathers as overall more intelligent than did female participants. In Furnham, Arteche, Chamorro-Premuzic, Keser, and Swami (2009) study, males also rated their fathers, but not their mothers as having higher scores for most intelligence types than did females. Yuen and Furnham (2006) reported higher male's estimates than those given by females, for several fathers' intelligence types and for mothers' spiritual intelligence. However, Furnham, Rakow, Sarmany-Schuller, and De Fruyt (1999) found out that Belgian males rated their fathers' overall IQ higher than Belgium females, but the opposite pattern appeared in a sample of Slovakian students. In addition, in Furnham and Wu (2008) study, gender differences occured for fathers' spatial and intrapersonal intelligence where females ascribed higher estimates than did male students. These findings indicate that raters’ gender may influence estimates of others, but its role and direction must be further explored and clarified.

## The Present Study

The goal of this study was to examine: i) twins’ agreement in their assessments of parental intelligence and behavioural genetic analysis of those ability estimates; ii) gender differences in SAI; and iii) raters’ and targets’ gender effects on parental assessments of intelligence.

As far as we know, none of the previous studies of parental intelligence estimates have included two raters from the same family and this is the first study of parents' intelligence estimation where sample consists of twin pairs. Due to this specific sample, we could investigate the rater-rater agreement of parental intelligence estimations by examining twins’ correlations in judgement of their parents' IQ scores. Since rater-rater agreement in judging the target traits depends on the level of knowing the targets and on the availability of the relevant cues which might be used for the accurate estimate of the trait (Funder, 1995), we expect substantial correlations between twins. Additionaly, by comparing MZ and DZ similarity and testing the behavioural genetic models, we could explore the aethiology of individual differences in impressions about the targets’ intelligence. Estimates of the genetic and environmental contribution to the individual differences in SAI based on this sample is published elsewhere (Bratko et al., 2012) and the result of that study suggests a substantial genetic effect. However, individual differences in twin assessments of parental traits reflect their impressions and, since both raters are rating the same target, the differences in similarity between MZ and DZ twins are not expected. Therefore, we expect that behavioural genetic model fitting of parental intelligence estimates would, in addition to always present non-shared environmental influence (E), reflect shared environmental influence (C) which primarily comes from the exposure to the target’s behaviour and from the availability of the relevant cues which are used to estimate parental intelligence.

The second goal of this study was to extend previous findings on gender differences in self-assessed overall and domain specific intelligences to a new culture. To date, there have been numerous studies in countries all over the world, e.g. in Argentina (Furnham & Chamorro-Premuzic, 2005), South Korea (Kang & Furnham, 2016), Egypt (Furnham & Mottabu, 2004), Iran (Furnham et al., 2012), Russia (Furnham & Shagabutdinova, 2012), Britain and Turkey (Furnham et al., 2009), etc. Most of these studies confirmed hubris-humility effect in SAI and showed that men perceive themselves higher on overall intelligence than women. Gender differences were also found in specific abilities, mostly on spatial and logical-mathematical where men provided higher estimates, while there was a tendency for women to have higher estimates on verbal/linguistic and personal intelligences, perceived as abilities that are more feminine. Although several studies included participants from European countries, none of them investigated gender differences in intelligence estimation on the Croatian sample. Thus, our next hypothesis is that man will have higher estimates than women on overall intelligence, as well as on more masculine abilities (spatial, logical-mathematical and body-kinesthetic intelligence), while women will have higher self-estimated scores on more feminine abilities (inter- and intra- personal, verbal/linguistic and musical intelligence). In addition, using available data on twins’ measured intelligence and personality, we expect that gender effect in SAI would not change when these variables are controlled for.

Along with gender differences in SAI, we were further interested in testing gender effect in twin estimations of parental intelligence, that is whether our participants would give higher intelligence scores to their mothers or to their fathers. Previous studies mostly showed that fathers are perceived as more overall intelligent than mothers. As with self-estimations, there is also an evident distinction between more masculine and feminine abilities in parental estimations. Fathers are perceived as more intelligent in the spatial and logical-mathematical domain, while mothers are seen more intelligent in the personal and musical domain. This pattern of result was confirmed in various countries, such as Argentina (Furnham & Chamorro-Premuzic, 2005), Iran (Furnham et al., 2012), Britain and France (Swami, Furnham, & Zilkha, 2009), etc. In accordance with those findings, we hypothesize that Croatian participants will give higher overall intelligence scores to their fathers than to their mothers and that fathers will be given higher scores on masculine types of abilities (spatial, logical-mathematical and body-kinesthetic intelligence), while mothers will be given higher scores on feminine types of abilities (personal, verbal/linguistic and musical intelligence).

Finally, we are aiming to investigate the effect of raters’ gender on parental estimates of intelligence by examining whether male and female participants differ in size of IQ scores given to their parents. As stated earlier, although the results from previous studies were inconsistent, some of them showed that men tend to give higher scores than women on overall and some specific intelligence types when estimating others (e.g. Swami et al., 2009, Furnham et al., 2001). Along with those findings, our final hypothesis was that there will be an effect of rater’s gender in a way that male participants will tend to give higher intelligence scores to their parents than female participants. For the interaction effect we did not have any specific expectations. Due to the multiple testing, we set the risk level for the acceptance of all hypotheses to one percent.

## Method

### Participants

Participants in the study were twins from the Zagreb area. The initial sample was formed in 2007 based on the register of citizens from which twin pairs born between 1985 and 1992 were identified. In total 2005 individuals were contacted and 732 (36.5%) returned filled in questionnaires via mail. From those individuals, 518 had data on self-assessed intelligence (79 MZ twin pairs, 83 DZS twin pairs, 82 DZO twin pairs and 30 twins without co-twin participating) and 483 participants had valid data of parents’ intelligence estimations. There were 220 male and 298 female participants, and their age varied between 15 and 22 years (*M* = 18.77, *SD* = 2.28).

### Measures and Procedure

#### Zygosity

Zygosity was determined with an 11-items questionnaire evaluating physical similarities (e.g. facial appearance, hair colour) and twin confusion by parents, other family members, teachers, casual friends and strangers. The use of questionnaires for zygosity determination has been shown to be accurate around 95% in different populations and cohorts (e.g. Torgersen, 1979; Reed et al., 2005; Song et al., 2010).

#### Self-Assessed Intelligence

We used a standard procedure to measure self-assessed intelligence. On one page a normal distribution was shown with a mean of 100 and distribution of six standard deviations (–3 to +3) together with brief descriptions of the anchor scores (e.g., 55 “mild retardation,” 100 “average,” 145 “gifted”). Below the distribution a grid with 11 rows and four columns was shown. In rows short descriptions were provided for overall intelligence and 10 multiple intelligences (verbal/linguistic, logical-mathematical, musical, body-kinesthetic, spatial, interpersonal, intrapersonal, naturalist, spiritual, and existential) taken from Gardner (1999). After reading these descriptions, participants were asked to write in columns estimates of their own, their mothers’ and their fathers’ intelligence. Self- and parental estimates for overall intelligence and eight multiple intelligences (verbal/linguistic, logical-mathematical, musical, body-kinesthetic, spatial, interpersonal, intrapersonal, and naturalistic intelligences) according to the Gardner’s (2006) theory were used in this study.

#### Measured Intelligence and Personality

Since SAI may partly reflect individual differences in real (measured) psychometric intelligence and personality, we have used these measures as control variables when testing the gender effect on overall SAI. Intelligence was assessed by the 20- itmes Croatian adaptation of a synononyms/antonyms subtest of General Aptitude Test Battery (Tarbuk, 1977) which is, according to the test manual, highly saturated with *g* factor. Personality traits were measured with Croatian adaptation of NEO-Five Factor Inventory (Costa & McCrae, 1992). The Cronbach's α coefficients were .81, .72, .57, .66, and .81 for Neuroticism, Extraversion, Openness, Agreeableness, and Conscientiousness, respectively.

## Results

### Rater Agreement and Behavioural Genetic Analysis of Parental Estimates of Intelligence

Due to the specific composition of our sample, we were able to examine if there is rater agreement for parental estimates of intelligence. We calculated twin intraclass correlations for intelligence estimates of mothers and fathers. As can be seen from Table 1, there is a significant agreement between twins in their ratings of both their fathers’ and mothers’ intelligences. Correlations range from .30 to .64 for fathers’ estimates, and .29 to .59 for mothers’ ability estimates. Rater agreement for both fathers’ and mothers’ estimates of intelligence is highest (all *r*s > .50) for overall intelligence, verbal/linguistic intelligence, logical-mathematical intelligence and interpersonal intelligence. The correlations of parental intelligence estimates were similar for MZ and DZ twins. The average correlations of MZ and DZ twins for estimation of overall parental intelligence was .63 and .56, respectively. 99% confidence interval around MZ and DZ twin correlations overlap indicating that there are no statistical differences between them. The twin agreement for fathers and mothers overall IQ estimate was .63 and .54, respectively, while the median estimate for eight Gardner’s specific abilities was .49, confirming the second set of hypotheses regarding substatial rater-rater agreement of parental estimates of intelligence. The average overall correlation of parental intelligence estimates across all twin pairs and both parents was .60^i^.

In addition, we ran genetic analyses on parental overall intelligence estimates in the statistical program Mx (Neale, Boker, Xie, & Maes, 2006; Neale & Maes, 2004). After assumption testing, univariate models were fitted in order to estimate A (additive genetic), C (common environmental) and E (unique environmental) influences, and then nested reduced univariate models (AE, CE and E) were compared to the full models in order to test which parameters were significant. For both fathers and mothers, confidence interval for the genetic influences (A) in the full model included zero and therefore A parameter could be dropped from the model. The best fitting model for both fathers’ and mothers’ intelligence estimates was a CE model with common environment (C) accounting for 63% and 51% of the variance, respectively.

**Table 1:** Twin Intraclass Correlations

Ability	Fathers as target	Mothers as target	Fathers as target		Mothers as target	
	All twins raters	All twins raters	MZ twins raters	DZ twins raters	MZ twins raters	DZ twins raters
Overall IQ	.64 (.53-.73)	.54 (.42-.65)	.65 (.43-.79)	.64 (.50-.75)	.61 (.39-.76)	.51 (.35-.64)
Verbal/Linguistic	.57 (.44-.67)	.59 (.47-.69)	.69 (.49-.82)	.52 (.36-.66)	.57 (.34-.74)	.60 (.45-.71)
Logical-mathematical	.62 (.51-.72)	.56 (.44-.66)	.53 (.28-.72)	.65 (.51-.75)	.72 (.55-.84)	.48 (.31-.62)
Musical	.42 (.27-.55)	.50 (.37-.61)	.41 (.13-.63)	.43 (.24-.58)	.60 (.37-.75)	.46 (.28-.60)
Body-kinesthetic	.31 (.15-.46)	.30 (.15-.45)	.37 (.09-.60)	.29 (.09-.47)	.50 (.25-.69)	.22 (.02-.40)
Spatial	.45 (.31-.58)	.42 (.27-.55)	.49 (.23-.69)	.44 (.25-.59)	.49 (.23-.68)	.38 (.20-.54)
Interpersonal	.62 (.49-.71)	.53 (.39-.64)	.69 (.48-.82)	.60 (.45-.72)	.53 (.28-.71)	.52 (.36-.66)
Intrapersonal	.49 (.35-.61)	.42 (.27-.54)	.51 (.26-.70)	.48 (.30-.62)	.54 (.30-.72)	.37 (.18-.53)
Naturalistic	.30 (.13-.45)	.29 (.14-.44)	.41 (.13-.63)	.24 (.04-.43)	.49 (.23-.68)	.21 (.01-.39)
*n* of pairs	228	242	73	155	78	164

*Note*. With 99% Confidence Intervals in parentheses.

### Gender Differences in Self-Assessed Intelligence

In order to examine if men and women differ in their self-assessed intelligence, we calculated a series of *t*-tests for independent samples. Since our sample consisted of twin pairs, we corrected the degrees of freedom associated with *t*-test values to half, so that the degrees of freedom associated with the calculated *t*-test values were 258 instead of 516, reflecting the number of pairs rather than number of individual participants (see McGue, Bacon, & Lykken, 1993 for detail explanation of that procedure). Also, Levene’s test for equality of variances was found to be violated for overall intelligence, *F*(1, 516) = 21.92, *p* < .001, logical-mathematical intelligence, *F*(1, 516) = 10.81, *p* = .001, and spatial intelligence, *F*(1, 516) = 8.34, *p* = .004). Owing to these violated assumptions, the *t*-statistic not assuming homogeneity of variance was computed for those intelligences. Results are presented in Table 2.

**Table 2:** Descriptive Statistics and Mean Differences in Self-Assessed Intelligence Between Males (n = 220) and Females (n = 298)

Intelligence	Males		Females		*t*	*df*	*d*
	*M*	*SD*	*M*	*SD*			
Overall IQ	108.99	12.72	105.17	9.54	3.74*	194.59	.34
Verbal/Linguistic	103.23	12.72	104.40	10.97	-1.12	258	-.10
Logical-mathematical	105.26	14.69	100.93	13.04	3.48*	219.22	.31
Musical	102.20	16.82	102.43	15.64	-0.16	258	-.01
Body-kinesthetic	114.54	16.29	108.41	14.85	4.46*	258	.39
Spatial	111.47	15.18	104.62	12.87	5.41*	212.40	.49
Interpersonal	110.51	13.53	111.77	12.86	-1.08	258	-.10
Intrapersonal	109.33	13.55	108.11	13.71	1.00	258	.09
Naturalistic	102.83	11.72	102.25	11.31	0.57	258	.05

*p < .01.

The results of statistical testing confirmed the hypothesis that male participants would have higher means on self- assessed overall intelligence and masculine abilities, namely logical-mathematical, body-kinesthetic, and spatial abilities. However, the hypothesis that females would have higher means on feminine abilities, namely inter- and intra- personal, verbal/linguistic, and musical intelligence was not confirmed.

Point-biserial correlation of the gender (male coded as 1) with the measured intelligence, Neuroticism, Extraversion, Openness, Agreeableness, and Conscientiousness were, respectively, .11, .29, .00, .07, .05, and .07. (*p* < .001 for Neuroticism, all other *r*s non significant). We calculated the correlation between gender and SAI before and after controlling for measured IQ and personality traits. These correlations were the same. To be more specific correlation between gender and self-estimated intelligence was *r*(518) = -.17, *p* < .001, while the partial correlation after controlling for measured IQ and personality traits was *r*(492) = -.17, *p* < .001.

### Effect of Raters’ and Targets’ Gender in Parental Estimates of Intelligence

In order to examine if people give higher estimates of intelligence to their fathers than to their mothers and if there are effects of raters’ gender on parental estimates of intelligence, we ran a series of analysis of variance. We tested the main effects of parents’ gender and raters’ gender on parental estimates of intelligence, as well as the interaction between parents’ and raters’ gender. Descriptive statistics for estimations of fathers’ and mothers’ intelligences, as well as for male and female raters separately, are presented in Table 3, and the results of the analyses of variance are presented in Table 4.

**Table 3:** Descriptive Statistics in Parental Estimates of Intelligence

Intelligence	Male raters				Female raters				Total			
	Fathers as target		Mothers as target		Fathers as target		Mothers as target		Fathers as target		Mothers as target	
	*M*	*SD*	*M*	*SD*	*M*	*SD*	*M*	*SD*	*M*	*SD*	*M*	*SD*
Overall IQ	111.58	13.25	108.69	11.17	108.19	11.93	105.87	10.81	109.62	12.60	107.05	11.04
Verbal/Linguistic	108.64	14.18	108.78	13.60	105.79	14.02	108.82	12.61	106.99	14.14	108.81	13.02
Logical-mathematical	110.39	15.83	102.88	12.75	109.74	14.50	102.72	12.36	110.01	15.06	102.79	12.51
Musical	100.03	13.86	101.18	11.89	99.72	13.61	99.02	13.22	99.85	13.70	99.93	12.71
Body-kinesthetic	106.45	14.65	100.15	12.81	105.69	12.57	100.36	11.64	106.01	13.47	100.27	12.13
Spatial	113.15	14.27	105.07	13.39	110.84	13.95	103.48	12.57	111.81	14.11	104.15	12.93
Interpersonal	105.82	15.25	112.26	13.51	103.35	14.13	108.98	14.01	104.39	14.65	110.36	13.88
Intrapersonal	108.87	13.35	109.44	13.12	104.59	13.39	107.45	13.01	106.39	13.52	108.29	13.08
Naturalistic	105.09	11.87	104.59	11.07	104.28	12.51	104.51	10.94	104.62	12.24	104.54	10.98
*n* of pairs	203	203	203	203	280	280	280	280	483	483	483	483

**Table 4:** Results of Analysis of Variance for Gender and Rater Effect in Parental Estimates of Intelligence (N = 483)

Intelligence	Targets’ gender (*F*)	Effect size ($\eta_{p}^{2}$)	Raters’ gender (*F*)	Effect size ($\eta_{p}^{2}$)	Targets x Raters (*F*)	Effect size ($\eta_{p}^{2}$)
Overall IQ	28.17*	.06	10.31*	.02	0.33	.00
Verbal/linguistic	5.68	.01	1.77	.00	4.71	.01
Logical-mathematical	92.09*	.16	0.16	.00	0.10	.00
Musical	0.11	.00	1.51	.00	1.83	.00
Body-kinesthetic	87.95*	.16	0.08	.00	0.61	.00
Spatial	109.52*	.19	3.78	.01	0.25	.00
Interpersonal	65.37*	.12	7.10*	.02	0.30	.00
Intrapersonal	10.84*	.02	8.11*	.02	4.82	.01
Naturalistic	0.07	.00	0.22	.00	0.51	.00

*Note. F* = *F* test for the effect of gender of a parents (targets), twins (raters) or targets x raters interaction; $\eta_{p}^{2}$ = partial eta squared for the effect size.

*p < .01.

None of the interactions was statistically significant. For the offspring estimates of parental overall intelligence, logical-mathematical intelligence, body-kinesthetic intelligence and spatial intelligence we found main effects of parents’ gender - fathers were given higher intelligence scores than mothers – mirroring the findings on self-estimates and supporting the gender effects on overall and masculine types of intelligence. Mothers were given the higher estimates for interpersonal and intrapersonal intelligence, so the set of hypotheses regarding feminine types of intelligence was partially confirmed. Significant effect of the raters’ gender was found for the estimates of parental overall intelligence, *F*(1, 481) = 10.31, *p* = .001, $\eta_{p}^{2}$ = .02, interpersonal intelligence, *F*(1, 481) = 7.10, *p* = .008, $\eta_{p}^{2}$ = .02, and intrapersonal intelligence, *F*(1, 481) = 8.11, *p* = .005, $\eta_{p}^{2}$ = .02, with females giving lower parental estimates than males. These findings partially confirmed the hypotheses about raters’ effects.

## Discussion

The goal of this study was to explore the raters’ agreement and the effect of raters’ and targets’ gender on self- and parental intelligence assessments in the sample of Croatian twins. This is the first twin study of parental intelligence assessment, and additionally, to our knowledge, the first study that uses more than one rater from the same family assessing intelligence of the other family member.

### Twin Agreement and Behavioural Genetic Analysis of Parental Estimates of Intelligence

First aim of this study refers to participants’ estimations of their parents' score on overall and eight Gardner’s intelligences. Due to our specific sample that consists of twin pairs, we had two raters from the same family, i.e. two independent intelligence estimates for mothers and fathers. Therefore, we were interested in investigating consensus between raters in estimation of their mothers’ and fathers’ intelligence scores. Results showed that all twin intraclass correlations were substantial. The twin correlation was .60 for the overall IQ estimate, and for the eight multiple intelligence estimates the median correlation was .49. The twin agreement for the parental overall intelligence estimate was higher than meta-analyzed mean correlation of two raters from the same family who judged Five-facor personality traits which ranged from .25 to .45 (Connelly & Ones, 2010), and higher than typical correlations between self- and other- intelligence assessments with measured intelligence (Ackerman & Wolman, 2007; Borkenau & Liebler, 1993; Bratko et al., 2012; Furnham, 2001), which are in the neigborhood of .30. However, obtained twin agreement was only slightly higher than correlation between self- and close acquaintance intelligence assessment (Borkenau & Liebler, 1993). Higher correlations between two impressions (self- rater or rater-rater assessments) than self- or rater- correlations with measured intelligence sugest that both SAI and OAI are, besides measured intelligence, based on the additional cues beyond pure behavioural expression of the ability. Similar twin correlations were found for both mothers’ and fathers’ intelligence estimates. However, the lowest agreement between raters was found for naturalistic (*r*s = .30 for fathers; *r*s = .29 for mothers) and body kinesthetic (*r*s = 31. for fathers; *r*s = .30 for mothers) intelligences, which may reflect the lower validity of these concepts from the Gardner multiple intelligence theory. On the other hand, the highest agreement was found for overall, logical-mathematical, interpersonal and verbal/linguistic intelligences. It can be assumed that the descriptions of those intelligence types were easier to understand and that raters had similar idea about their meaning. Furthermore, since the highest consensus was found for overall intelligence, it seems that raters were having a similar definition and view of what general intelligence is.

Since to our knowledge there are no twin studies of OAI, we also used the specific compostion of our sample to examine genetic and environmental influences to individual differences in OAI. MZ and DZ correlations for both mothers’ and fathers’ overall intelligence scores were similar and model fitting has indicated that individual differences in OAI can be contributed to common and unique environmental influences. This is different from the usual pattern where genetic factors play an important role in explaining individual differences in a trait. However, substantial effect of the common environment indicates that twin judgments of parental intelligence are not genetically mediated and are guided by the cues, which come from the exposure to the same environment.

### Gender Differences in Self-Assessed Intelligence

The second goal of this study was to extend the existing findings on gender differences in assessing own intelligence to a new culture and with a specific twin sample. Most of the obtained results confirmed our hypotheses showing that the universal pattern of gender differences in SAI can also be found in a Croatian sample. We wanted to investigate if male and female participants differ in self-assessed overall IQ and eight domains of intelligence defined by Gardner (2006). In accordance with the most of previous studies in the area, gender differences in SAI were found for overall intelligence, logical-mathematical intelligence, body-kinesthetic intelligence and spatial intelligence, with males reporting higher SAI scores on these domains compared to females. This is completely in line with our hypothesis that men will have higher estimates than women on overall intelligence, as well as on those abilities that are usually perceived as more masculine. The biggest effect was found for spatial intelligence (*d* = .49), very similar to the effect size (*d* = .43) reported in a meta-analytic study of gender differences in SAI by Syzmanowicz and Furnham (2011). Since findings support that there are real differences between men and women in spatial ability (e.g. Voyer et al., 1995), we can assume that gender difference in SAI on spatial domain reflects this difference that really exists between genders. On the other hand, females have not perceived themselves significantly higher than males in any intelligence domain. We have not found gender differences in either personal, musical or verbal ability, as hypothesized since those domains are stereotypical perceived as more feminine. However, in some of the studies female participants also did not provide significantly higher results in any intelligence domain (e.g. Furnham & Chamorro-Premuzic, 2005; Furnham & Wu, 2008; Kang & Furnham, 2016), including the study in Poland (Furnham, Wytykowska, & Petrides, 2005). Therefore, we can say our results indicate existence of hubris-humility effect in a Croatian sample, where male participants are prone to give themselves higher IQ scores than females on overall and few specific abilities, while female participants do not have that pattern in any intelligence domain. In addition, our results are in line with the hypothesis that culture plays a role in hubris-humility effect. Storek (2011) found that the effect size (η^2^ = .08) for mathematical and spatial intelligence or domain-masculine IQ was in Czech Republic smaller than in 6 UK samples (η^2^ range = .17-.32). In our study effect size for mathematical intelligence was η^2^ = .05, and for spatial intelligence η^2^ = .13. However, it is important to point out that both males’ and females’ average scores on overall as well as on each of eight types of intelligence is above the population theoretical mean score of 100. That is in line with findings that people generally have a tendency to overestimate their abilities, known as ''lake Wobegon'' effect (Kruger, 1999). In our study, males gave themselves the highest estimates on body-kinesthetic intelligence (*M* = 114.54, *SD* = 16.29), while females scores were highest on interpersonal intelligence (*M* = 111.77, *SD* = 12.86).

### Effect of Raters’ and Targets’ Gender in Parental Estimates of Intelligence

Besides participants’ agreement in their judgment of parents’ intelligence, we wanted to investigate the effect of both raters’ and targets’ gender in the assessment on parental intelligence estimation. We have found main effects for parents’ gender and raters’ gender in parental estimation but no interaction effects. Results of the main effects of parental gender were in line with our hypothesis. Fathers were estimated as more intelligent than mothers on same domains where male participants provided higher SAI scores - on overall intelligence, logical-mathematical intelligence, body-kinesthetic intelligence and spatial intelligence. On the other hand, on interpersonal intelligence and intrapersonal intelligence participants gave higher intelligence scores to mothers than to fathers. The biggest effect was found for spatial intelligence ($\eta_{p}^{2}$ = .19) followed by logical-mathematical and body-kinesthetic intelligence ($\eta_{p}^{2}$ = .16). These results are correspondent with Bennett's (2000) idea that different intelligence types can be divided into two factors – masculine and feminine abilities, and it seems that those stereotypic views are present among Croatian participants. However, our hypothesis was not fully confirmed because mothers were not perceived as more intelligent in verbal/linguistic or in musical domain. Despite the fact that verbal intelligence is traditionally seen as a female type of ability, it is interesting that gender difference in parental estimation of that domain was not found in some of the other studies as well, and in some of them fathers were given even higher scores (Furnham et al., 2012; Rammstedt & Rammsayer, 2000; Swami et al., 2009). The crucial question is why gender differenes in estimates of parental intelligence exist? If the assumption that OAI depends on additional cues, besides real gender differences in ability, is correct, then we can ask ourselves which cues judges use when they assess parental intelligence.

One possible cue to higher intelligence of fathers than mothers could be the divison of housework and family decision making. Research has shown that women are still responsible for about two thirds of routine housework (Bartley, Blanton, & Gilliard, 2005; Bartolac & Kamenov, 2013; Lachance-Grzela & Bouchard, 2010). Davis (2010) points that one of the main reasons why women do more housework than men is that they have less power. Furthermore, individual with more power in the relationship makes more decisions in that relationship (Fox & Murry, 2000). An interesting study by Zipp, Prohaska, and Bemiller (2004) tested if the asymmetry between husbands and wives on decision making, the division of household labor, child care, and so forth can be explained, in part, by taking into account the invisible power of men. Results showed that wives were much more likely than husbands to agree with their spouses’ known answers, and that tendency was virtually unaffected by women’s financial, cultural, and political capital.

The other possible cue to higher intelligence of fathers than mothers could be related to working positions and earnings. Recent gender gap report (World Economic Forum, 2018) has shown that there are more women than men in education and health industry, while the largest industry gender gaps were found for manufacturing, energy and mining, and software and IT services industry with more men than women. In addition, it was found that male professionals are found in positions that are generally more lucrative and of a more senior level. In line with that, income gaps are particularly persistent, not only in pay, but also in division of economic power. Longitudinal data from men and women who were top 1% in mathematical reasoning ability at age 13, showed that even in women and men of similar abilities, men earned more than women later in life, and were more likely to be chief executives and employed in information technology and STEM positions (Lubinski, Benbow, & Kell, 2014).

Finally, our analysis showed that there were also effects of raters' gender on some parental estimates. Male participants gave higher estimates than females to their parents on overall intelligence, interpersonal intelligence and intrapersonal intelligence. Females did not give significantly higher scores in any domain. Although the results of raters' gender effect were not always consistent through studies, those that reported differences in how genders estimated others usually showed that men were more prone to give higher IQ scores than women (Furnham et al., 2001; Swami et al., 2009). However, it is important to note that in our study differences were found in only three estimated domains and all of those effect sizes were relatively small ($\eta_{p}^{2}$ = .02).

### Limitations and Conclusion

The conducted study of twins’ self- and parental intelligence estimates yielded several relevant findings, but also has some limitations which can be taken into account when planning future research. Firstly, the twins’ mean correlation in their assessments of overall parental intelligence was substantial and we can rarely see rater-rater correlations, which are so high when judging the target traits. That correlation is higher than correlations of SAI and OAI with psychometrically tested intelligence. Thus, it may indicate the possibility that people use additional cues beyond intelligence in their subjective estimation of that trait. The future research migh be directed toward the identification of these cues. Behavioural genetic analysis of the assessed parental intelligence yielded substantial shared environmental effect indicating that these cues obviously lie in the same environment to which the twins are exposed.

Secondly, it is obvious that twin agreement is substantially higher for overall intelligence than for the Gardner’s multiple intelligences. That is especially visible for the naturalistic and body kinaesthetic intelligence, which are probably difficult to judge for the raters. However, this may also indicate the lower validity of these Gardner’s theoretical concepts. Thus, future research on sources of the individual differences in SAI and OAI may benefit from other theoretical frameworks. For example, some of the studies in the field used Sternberg’s three types of intelligence (Furnham et al., 2009; Kang & Furnham, 2016; von Stumm et al., 2009), Thurstone's seven primary mental abilities (Ortner et al., 2011; Rammstedt & Rammsayer, 2000), 10 broad abilities from Cattell-Horn-Carroll theory of intelligence (Furnham & Mansi, 2014; Kang & Furnham, 2016) or concept of emotional intelligence (Petrides et al., 2004; von Stumm et al., 2009).

Regarding gender effects, the expectations about males’ higher self-assessments for overall intelligence and for the masculine types of abilities were confirmed, while for the feminine types of abilities there were no significant gender effects. Both target and rater effects were found for the parental estimates of intelligence. One of the limitations of this research certainly lies in the fact that we only reported results on parental assessments of intelligence without parents’ scores on objective psychometric intelligence test. Thus, it is difficult to estimate to which extent obtained gender differences in other-assessments reflect the real individual differences in intelligence within the particular sample or the non-cognitive individual differences which might account for the observed parental differences. For SAI, the gender effect for the overall intelligence was controlled for the measured intelligence and personality. However, it should be noted that substantial (measured) gender differences are more probable for the specific abilities, and for those we do not have the control either for SAI or for the parental intelligence estimates. Future studies should therefore include both evaluations of the overall intelligence and specific abilities, as well as objectively measured general intelligence and specific abilities together with personality traits in order to test these different hypotheses.
